# Effect of Strain Rate on the Transverse Tension and Compression Behavior of a Unidirectional Non-Crimp Fabric Carbon Fiber/Snap-Cure Epoxy Composite

**DOI:** 10.3390/ma14237314

**Published:** 2021-11-29

**Authors:** Khizar Rouf, Aaditya Suratkar, Jose Imbert-Boyd, Jeffrey Wood, Michael Worswick, John Montesano

**Affiliations:** 1 Mechanical and Mechatronics Engineering Department, University of Waterloo, 200 University Ave W, Waterloo, ON N2L 3G1, Canada; krouf@uwaterloo.ca (K.R.); jmsimbertboyd@uwaterloo.ca (J.I.-B.); michael.worswick@uwaterloo.ca (M.W.); 2 Mechanical and Materials Engineering Department, Western University, 1151 Richmond Street N, London, ON N6A 5B9, Canada; asuratka@uwo.ca (A.S.); jtwood@uwo.ca (J.W.)

**Keywords:** non-crimp fabric composites, snap-cure epoxy, split Hopkinson pressure bar, dynamic testing, pulse shaping, transverse tension, transverse compression

## Abstract

The strain rate-dependent behavior of a unidirectional non-crimp fabric (UD-NCF) carbon fiber/snap-cure epoxy composite loaded along the transverse direction under quasi-static and dynamic conditions was characterized. Transverse tension and compression tests at quasi-static and intermediate strain rates were performed using hydraulic testing machines, while a split Hopkinson pressure bar (SHPB) apparatus was used for transverse compression tests at high strain rates. A pulse shaper was used on the SHPB apparatus to ensure dynamic equilibrium was achieved and that the test specimens deformed homogenously with a nearly constant strain rate. The transverse tensile strength at a strain rate of 16 s^−1^ increased by 16% when compared to that at quasi-static strain rates, while distinct localized fracture surface morphology was observed for specimens tested at different strain rates. The transverse compressive yield stress and strength at a strain rate of 325 s^−1^ increased by 94% and 96%, respectively, when compared to those at quasi-static strain rates. The initial fracture plane orientation for the transverse compression tests was captured with high-speed cameras and found to increase with increasing strain rate. The study provides an important data set for the strain rate-dependent response of a UD-NCF composite material, while the qualitative fracture surface observations provide a deeper understanding of the failure characteristics.

## 1. Introduction

Composite materials comprising heavy-tow non-crimp fabric (NCF) reinforcements are increasingly considered for use in primary load-bearing structures due to their excellent specific mechanical properties and relatively low cost when compared to other liquid composite molded materials reinforced with woven or braided fabrics. NCFs can be combined with a snap-cure resin to fabricate high-volume production automotive structural components with short cycle times [1]. Automotive structures experience loads under a range of quasi-static and dynamic loading conditions during service (typically <300 s^−1^); therefore, widespread adoption of NCF composites in automobiles requires a comprehensive understanding of their constitutive behavior at various strain rates [2].

Several researchers [2,3] have characterized the strain rate-dependent response of unidirectional (UD) tape composites and woven fabric composites, reporting that they exhibit strain rate dependency in their matrix-dominated deformation modes, such as transverse tension and transverse compression. Schaefer et al. [4] studied the transverse tensile strain rate-dependent response of IM7–8552 (58% fiber volume fraction), a toughened carbon/epoxy system, and reported a slight increase in the ultimate strength and strain-to-failure. The elastic modulus was found to be strain rate-independent. Gilat et al. [2] and Taniguchi et al. [5] investigated the effect of strain rate on the transverse tensile response of IM7/977–2 and T700S/2500 (67% fiber volume fraction) composite materials, respectively. A noticeable increase in the elastic modulus was reported for both materials, which contradicts the results from [4], while the strength was also reported to increase; however, the strain-to-failure was independent of strain rate. Koerber et al. [6] studied the effect of strain rate on the transverse compressive response of IM7/8552 and found a significant increase in the ultimate compressive strength and a notable increase in the elastic modulus with increase in strain rate. However, the strain-to-failure was found to be rate-independent. Schaefer et al. [4] observed an increase in the transverse compressive strength and modulus with the increase in strain rate for IM7/8552; however, a noticeable decrease in the strain-to-failure was reported. Yokoyama et al. [7] found a significant increase in the transverse compression strength and elastic modulus of the carbon fiber/epoxy material system T700/2521 (65% fiber volume fraction); however, a significant decrease in the strain-to-failure was observed, which contradicts the results reported in [6]. Another point of disagreement is the effect of strain rate on the initial fracture angle for the transverse compression tests. Weigand et al. [8] and Vural et al. [9] reported that there was no significant effect of strain rate on the fracture angle [2]. However, May et al. [10] observed a noticeable increase in the fracture angle with the strain rate.

Although several studies have focused on characterizing the strain rate-dependent response of UD tape and woven fabric composites, the reported trends for these material systems are inconsistent. These discrepancies may be due to the different manufacturing processes used in each study that can lead to variation in material quality or degree of cure for the matrix, the variation in matrix type among the studies, or due to variations with the testing protocol. Moreover, most studies have focused on characterizing the strain rate-dependent response of composites comprising standard thermosetting resins that require long curing cycles. The strain rate-dependent response of fiber-reinforced composites with snap-cure resins has not been studied to date. Moreover, the reported studies do not consider the strain rate dependency of composites with NCF reinforcements, which have distinct microstructures when compared to UD tape composites. NCF composites typically comprise distinct tows through their cross-section with light supporting fiber yarns and a polyester stitching [11,12], which results in resin-rich regions, in-plane tow misalignment, and crimping caused by compaction during fabrication [1], [13]. Thus, the effect of strain rate on the response of NCF composites may be distinct to that of UD tape composites.

The present work aimed to characterize the strain rate-dependent behavior of a UD-NCF carbon fiber/snap-cure epoxy composite material manufactured using a high-pressure resin transfer molding (HP-RTM) process. In-plane transverse tension and compression tests were conducted at quasi-static and dynamic loading rates to capture the deformation response and failure modes. High-speed cameras were used during intermediate and high strain rate tests to capture failure of the test specimens. The current study addresses a major gap in the literature by characterizing a high-performance automotive NCF composite for the first time, while establishing a suitable dynamic testing protocol.

## 2. Materials and Experimental Set-Up

### 2.1. Materials and Test Specimens

The studied composite material consisted of PX35 UD300 (Zoltek, St. Louis, MO, USA which is a heavy tow UD-NCF reinforcement. The fabric comprised straight tows each consisting of 50,000 continuous carbon fibers, supporting glass fiber yarns oriented perpendicular to the tows, and a light polyester stitching in a tricot pattern along the tow direction [11]. The areal density of the fabric was 333 g/m^2^, with the carbon fiber accounting for 92.8% of the total weight. The weight fraction and linear density of the glass fiber were 3.0% and 34 dtex, respectively, and, for the polyester stitching, were 1.8% and 76 dtex, respectively. The matrix was a highly reactive snap-cure three-part epoxy system (Hexion Inc.) comprising EPIKOTE^TM^ Resin TRAC 06150, EPIKURE^TM^ curing agent TRAC 06150, and the internal mold release agent HELOXY^TM^ Additive TRAC 06805, which were mixed with the ratio of 100:24:1.2 parts by weight, respectively [14]. Flat panels of the UD-NCF composite measuring 900 × 550 mm were manufactured using an HP-RTM process [1], which comprised either 7 or 11 layers of the fabric. Resin and hardener were mixed at high pressure of 120 bar and injected at high flow rate of 40 g/s into the preheated mold using a metering unit. The mold temperature was maintained at 120 ⁰C. A press force of 1500 kN was initially applied to the mold during injection and increased to 5500 kN during curing. The nominal thicknesses of the [0]_7_ and [0]_11_ panels were 2.8 and 4 mm, respectively. The fiber volume fraction of the fabricated panels was measured using optical microscopy and was approximately 45% for all panels [1]. Optical microscopy also revealed that the void content in the fabricated panels was negligible (<2%).

The specimens used for the transverse tensile and transverse compression tests were cut from the [0]_7_ and [0]_11_ panels, respectively, using an abrasive water jet cutting machine with an abrasive mesh size of 80 and a pressure of 30,000 psi. It was ensured that all test specimens had at least two unit cells of the fabric along the length and width directions, as recommended by the standard ASTM D6856 for textile composites [15]. Each test specimen was visually inspected to ensure that there no defects as a result of the cutting process.

### 2.2. Quasi-Static Transverse Tension Test Set-Up

**Figure 1:**
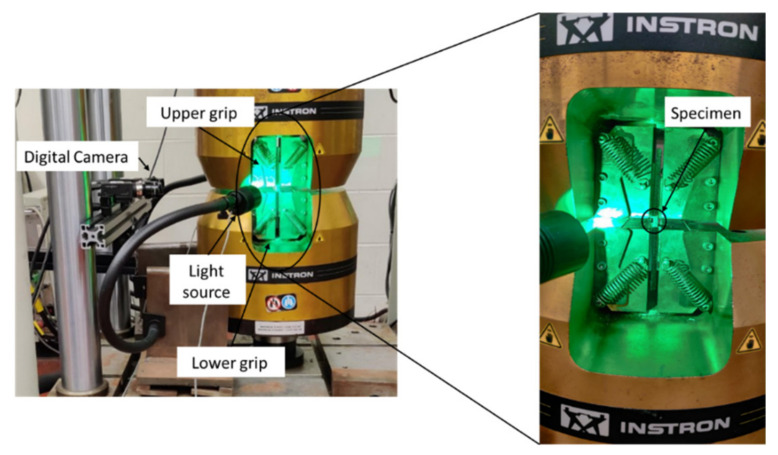
Experimental set-up for transverse tension and compression tests at quasi-static strain rates (compression test shown).

The quasi-static tensile tests were conducted in accordance with ASTM D3039 [16] using a servo-hydraulic test frame (Instron 8804, Norwood, MA, USA) (Figure 1), which consisted of a 250 kN load cell. All tests were performed under displacement control with a constant displacement rate of 2 mm/min. The rectangular test specimens measured 200 × 25 mm, and carbon fiber/epoxy composite end tabs were bonded to the specimens with Devcon 5 Minute^®^ Epoxy using a dispenser, resulting in a gauge length of 50 mm. Hydraulic wedge grips were used to grip the test specimens at the end tabs. The surface of the specimens was speckled with a white-on-black paint pattern to facilitate strain measurements using digital image correlation (DIC) software (VIC3D). Two GRAS-50S5M-C digital cameras (Point Grey) with 2448 × 2048 pixel resolution (0.02 mm/pixel × 0.012 mm/pixel) were used to capture images during the test at a frame rate of 3.33 frames/second.

### 2.3. Intermediate Strain Rate Transverse Tension Test Set-Up

**Figure 2:**
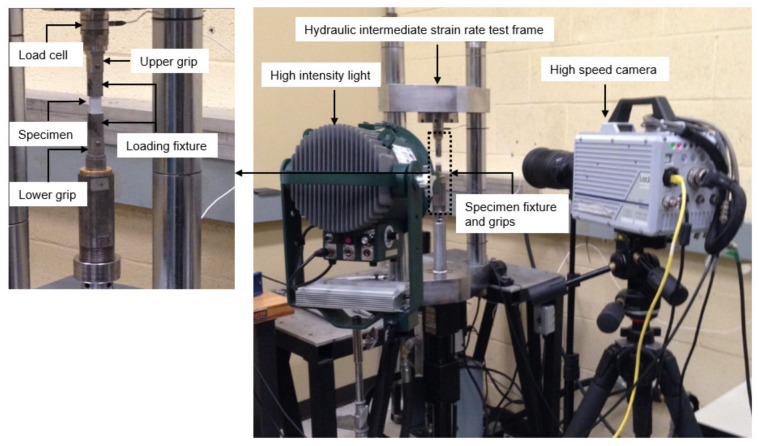
Experimental set-up for transverse tension tests at intermediate strain rates.

**Figure 3:**
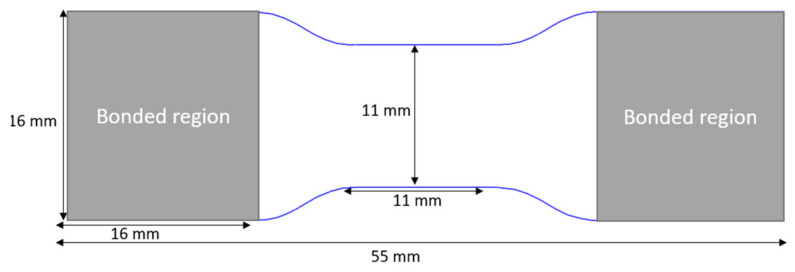
Specimen geometry for transverse tension tests at intermediate strain rates.

Transverse tensile tests at intermediate strain rates were conducted on a custom-built hydraulic test frame (Figure 2) comprising a 13.3 kN load cell and a hydraulic actuator with a 101.6 mm stroke. The upper specimen grip was mounted to the load cell on the stationary upper crosshead. The lower specimen grip was mounted to a fixture on the actuator piston, which utilized a slack adaptor to provide sufficient time for the hydraulic piston to accelerate to the desired speed before engaging the specimen. Dog bone specimens (Figure 3) were bonded to the grips using Loctite^®^ 480 adhesive, which is a one-part ethyl cyanoacrylate instant resin. All tests were performed under displacement control conditions at two loading rates, 20 mm/s and 1300 mm/s. Each test specimen was speckled with a black-on-white paint pattern to facilitate strain measurements using a DIC software GOM Correlate (GOM, Brunswick). The deformation response of the specimen surface was obtained from the images captured by a high-speed camera, FASTCAM SA-5 (Photron, Tokyo). The images were captured with image resolution of 256 pixel × 408 pixel (0.063 mm/pixel × 0.061 mm/pixel) at 10,000 frames/second and 54,250 frames/second for the 200 mm/s and 1300 mm/s tests, respectively.

Specimen thickness was 2.8 mm.

### 2.4. Quasi-Static Transverse Compression Test Set-Up

The same set-up described for the quasi-static transverse tension tests was used for the quasi-static transverse compression tests (Figure 1). The tests were performed in accordance with ASTM D3410 using a displacement rate of 1 mm/min. The rectangular test specimens had a nominal gauge length of 10 mm and a width of 25 mm. End tabs were bonded to the specimens to promote the occurrence of failure in the gauge length, as described in Section 2.2. The specimens were also speckled with a white-on-black paint pattern, and the same DIC set-up described in Section 2.2 was used.

### 2.5. Intermediate Strain Rate Transverse Compression Test Set-Up

**Figure 4:**
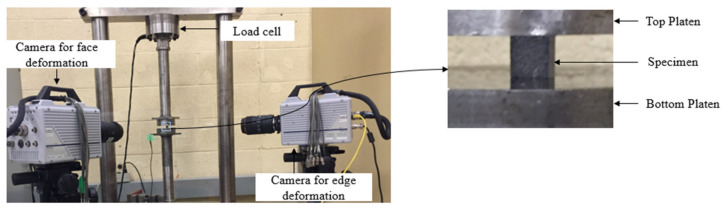
Experimental set-up for transverse compression tests at intermediate strain rates.

Intermediate strain rate transverse compression tests were performed using a custom hydraulic test frame (Figure 4), with a 25,000 lbf (111,206 N) load cell. Compression platens were used to compress the test specimens with a displacement rate of 2 mm/s. Each specimen was machined using a titanium carbide bit to ensure the loading ends were flat and parallel. The nominal dimensions of the test specimens were 10 × 10 mm. Before loading, a thin layer of a high-pressure grease (molybdenum sulphide) was applied to the loading ends of the specimens to reduce friction between the specimen and platens. Two FASTCAM SA-5 high speed cameras were used to capture the specimen deformation for subsequent strain measurement, one focused on the surface and the other on the edge of the specimen. A frame rate of 2,500 frames per second with an image resolution of 384 × 592 pixel^2^ (0.026 mm/pixel × 0.017 mm/pixel) was selected to capture the images for both cameras. The images obtained from the edge of the specimens were also used to compute the extent of out-of-plane bending and the fracture angle.

### 2.6. High Strain Rate Transverse Compression Test Set-Up

**Figure 5:**
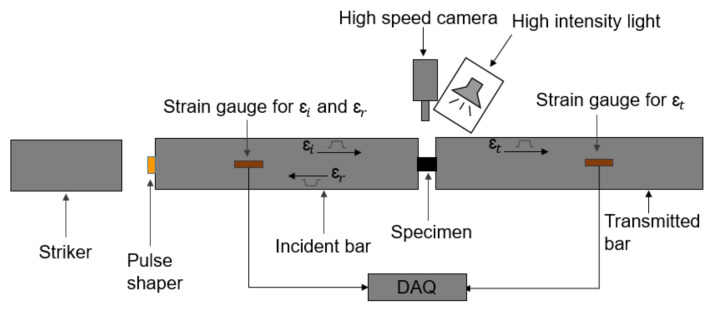
Schematic diagram of a compression split-Hopkinson pressure bar apparatus.

High strain rate transverse compression tests were performed on a compression split Hopkinson pressure bar (SHPB) apparatus, which consisted of a striker bar, incident bar, and transmitted bar (Figure 5). The test specimen was placed between the incident and transmitted bars. The striker bar was accelerated by compressed air using a gas gun to impact the incident bar, which generates a rectangular pulse that travels towards the non-striking end of the incident bar where the specimen is located. Once the pulse reaches the end of the bar, a portion of the pulse is transmitted through the specimen to the transmitted bar, while a portion is reflected back to the incident bar. The incident and reflected pulses are measured by the strain gauges, interconnected in Wheatstone bridge circuits, and mounted on the incident bar, while the transmitted pulse is measured by the strain gauges, interconnected in Wheatstone bridge circuits, and mounted on the transmitted bar (Figure 5). In SHPB analysis, the strain rate, stress, and strain of the tested specimens are obtained using the incident, reflected, and transmitted pulses recorded by the strain gauges. One-dimensional (1D) wave propagation theory [17] can be used to define the relevant parameters, which are given by

(1) $$\varepsilon_{s}=-\frac{2\times c}{l_{s}}\;\int_{0}^{t}\varepsilon_{R}dt$$

(2) $$\dot{\varepsilon_{s}}=-\frac{2\times c}{l_{s}}\;\varepsilon_{R}\left(t\right)$$

(3) $$\sigma_{s}=E\;\left(\frac{A_{b}}{A_{s}}\right)\times \varepsilon_{T}$$

Here, $\varepsilon_{s},\;\dot{\varepsilon_{s}},\;\sigma_{s},\;\varepsilon_{R},\;\varepsilon_{T},\;E,\;A_{b},\;A_{s},\;c,\;\mathrm{and}\;l_{s}$ represent the specimen strain, specimen strain rate, specimen stress, reflected strain in the incident bar, transmitted strain in the transmitted bar, modulus of the bars, area of the transmitted bar, area of the specimen, bar wave velocity, and specimen length, respectively.

**Figure 6:**
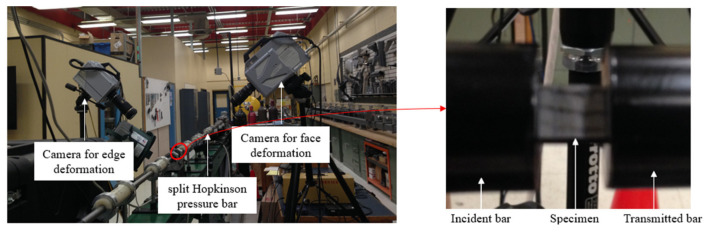
Setup for transverse compression tests at high strain rates using SHPB apparatus.

In this study, the SHPB apparatus consisted of 25.4 mm diameter maraging steel bars (Figure 6). The striker bar was 0.6 m long, while the incident and transmitted bars were each 2.4 m long. An oxygen-free high conductivity (OFHC) copper pulse shaper of 0.5 mm thickness and 7 mm diameter was used to achieve dynamic equilibrium and a nearly constant strain rate during the tests. The pulse shaper was identified on the basis of the pulse shaping analysis (PSA) proposed by Nemat-Nasser et al. [18].

Specimens were machined using a titanium carbide bit to ensure the loading ends were parallel, resulting in nominal dimensions of 10 × 10 mm. Each specimen was speckled with a black-on-white pattern to capture deformation for the strain measurements. Before loading, a thin layer of a high-pressure grease (molybdenum sulphide) was applied to the loading ends of the specimens to reduce friction between the specimen and bars.

Two high-speed cameras, a FASTCAM SA-Z and a FASTCAM SA-5, were used to track deformation of the specimen (Figure 6). The strains were measured using a DIC software, GOM Correlation, with images captured by the FASTCAM SA-Z camera with a frame rate of 240,000 frames per second and an image resolution of 256 × 184 pixel^2^ (0.039 mm/pixel × 0.054 mm/pixel). The FASTCAM SA-5 camera was used to capture the edge deformation using a frame rate of 100,000 frames per second and an image resolution of 320 × 192 pixel^2^ (0.031 mm/pixel × 0.02 mm/pixel).

**Figure 7:**
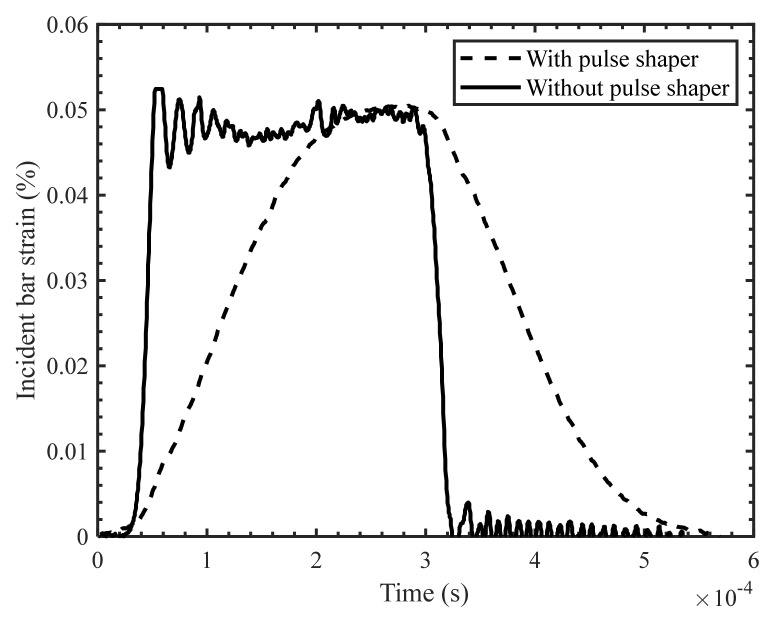
Incident bar strain pulse with and without pulse shaper.

Initial pulse shaping trials revealed that the copper pulse shaper adequately provided a ramp pulse shape (Figure 7). Ramp-shaped pulses provide greater rise time when compared to a conventional rectangular wave, which aids in achieving dynamic equilibrium and homogenous deformation of the specimens being loaded. Another advantage of using pulse shaper is removal of the unwanted oscillation in the signal (Figure 7).

## 3. Results and Discussion

### 3.1. Transverse Tension Tests

**Figure 8:**
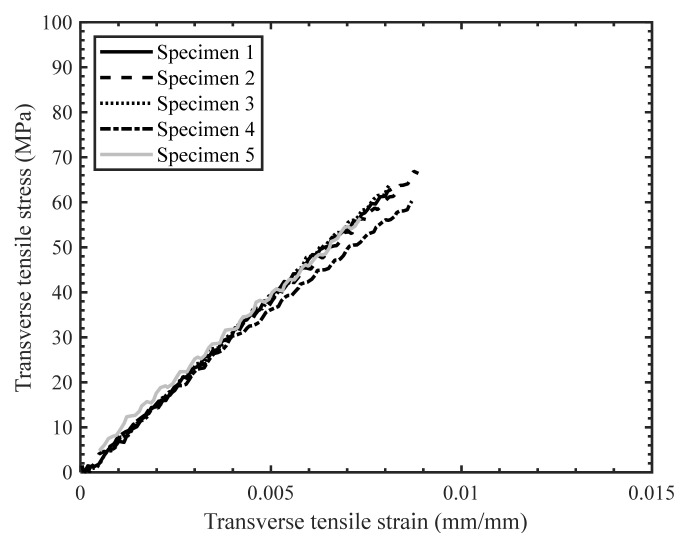
Stress-strain response for transverse tension tests with a strain rate of 2.2 × 10^−4^ s^−1^.

**Figure 9:**
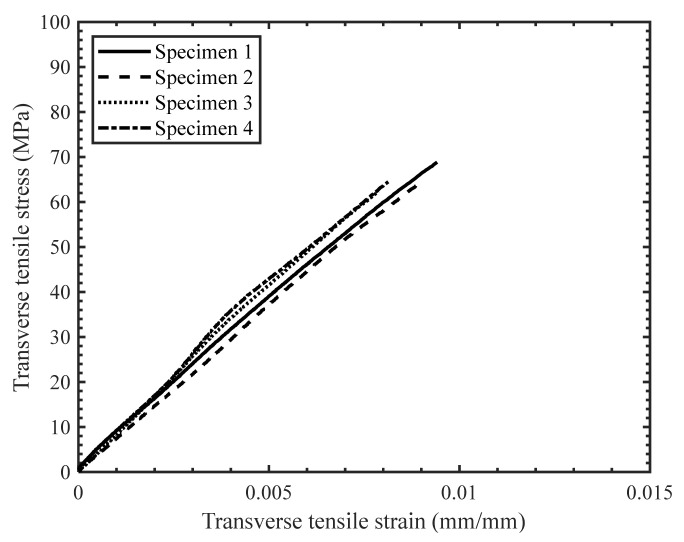
Stress-strain response for transverse tension tests with a strain rate of 0.1 s^−1^.

**Figure 10:**
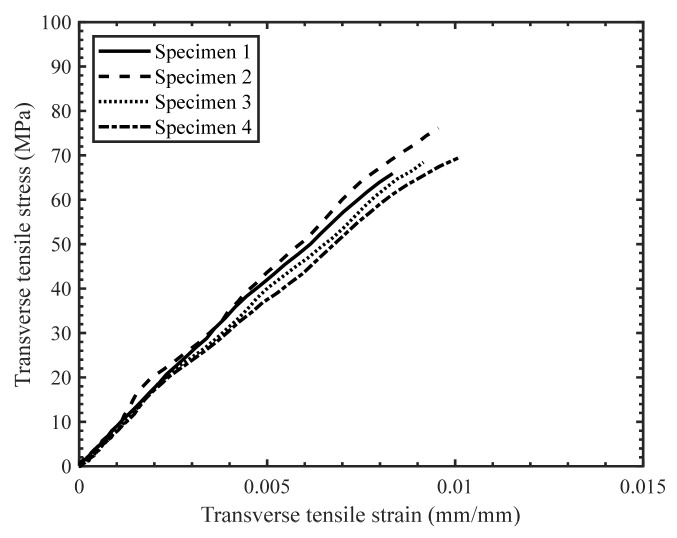
Stress-strain response for transverse tension tests with a strain rate of 16 s^−1^.

**Figure 11:**
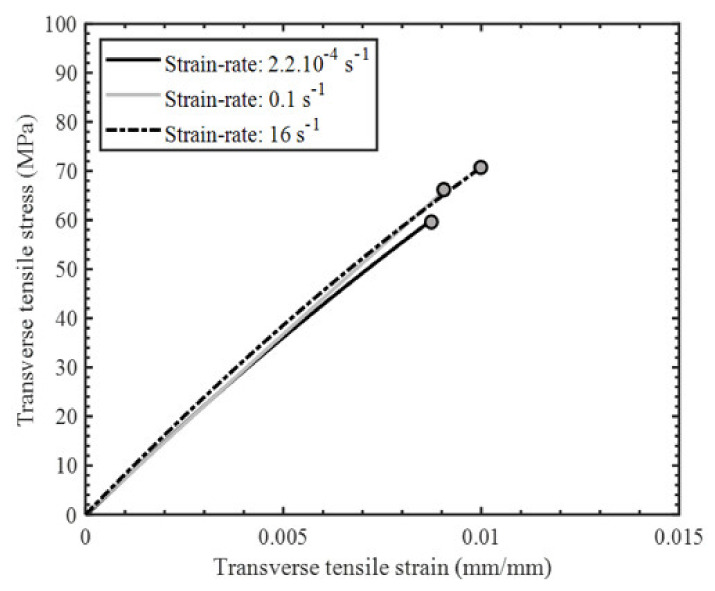
Representative transverse tension stress–strain response (second–order polynomial fit) for indicated strain rates.

**Figure 12:**
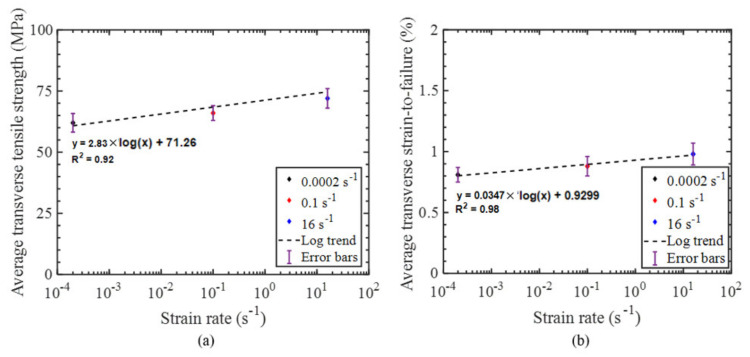
(**a**) Average transverse tensile strength versus strain rate, and (**b**) average transverse tensile strain–to–failure versus strain rate with regression lines shown.

The stress–strain response of the tested specimens for all strain rates was found to be repeatable with minor scatter (Figure 8, Figure 9 and Figure 10). Therefore, the extent of manufacturing-induced defects in the specimens (e.g., voids, thickness variations) and variations in the test set-up were deemed to be negligible. For all strain rates considered, the stress–strain response was nearly linear elastic with slight nonlinearity at increasing levels of strain, while all specimens failed at the center of the gauge section in a brittle manner. The measured properties for the considered strain rates are reported in Table 1. The strain-to-failure of the tested specimens was found to be within the range 0.75–1%, which is similar to that reported for carbon fiber/epoxy UD tape composites [5]. The average ultimate transverse tensile strength and strain-to-failure were found to increase by 16% and 20%, respectively, with an increase in strain rate from 2.2·10^−4^ s^−1^ to 16 s^−1^ (Figure 11). The mean increase in the elastic transverse modulus over the same range of strain rate was less than 5% and was considered to be negligible, having also been reported by Schaefer et al. [4] for a UD tape composite. Note that the transverse elastic modulus measured at 0.1 s^−1^ strain rate was lower than the modulus measured at 2.2·10^−4^ s^−1^ strain rate, while the standard deviation was also higher for the former. This result may be due to a slight variation in the tested specimens (e.g., quality or alignment of fibers with respect to the specimen axis). However, the difference between the elastic modulus obtained at both 2.2·10^−4^ s^−1^ and 0.1 s^−1^ strain rates was within one standard deviation, indicating that there was no statistically significant difference in the elastic modulus. The empirical relationships for the average transverse tensile strength and strain-to-failure as a function of strain rate were established by fitting the data with a semi-logarithmic expression (Figure 12), which can be used to interpolate these properties at other strain rates.

**Figure 13:**
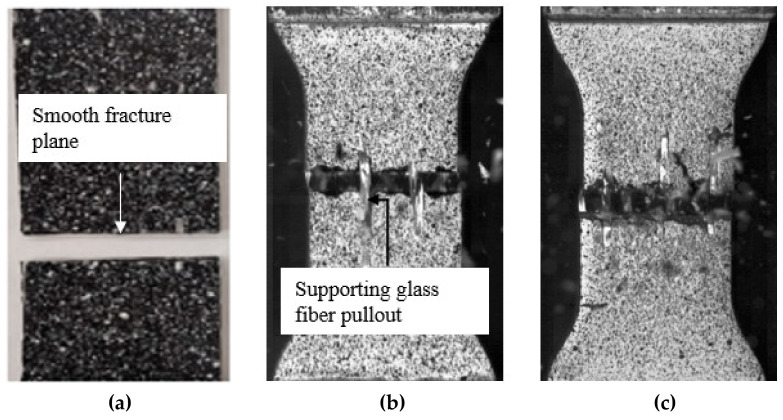
Images of failed transverse tension specimens at strain rates of (**a**) 0.0002 s^−1^, (**b**) 0.1 s^−1^, and (**c**) 16 s^−1^. Note that the images in (**b**,**c**) were obtained with a high-speed camera.

**Figure 14:**
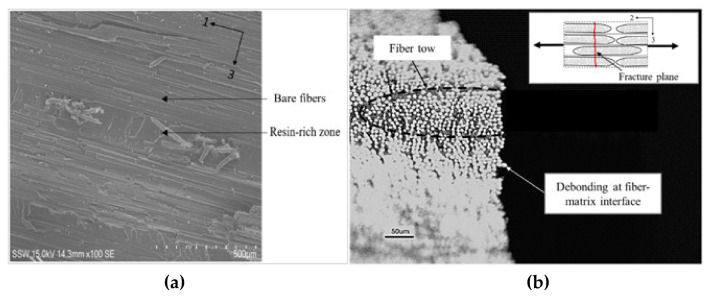
Quasi-static transverse tension test (strain rate 0.0002 s^−1^): (**a**) Scanning electron microscope image of the fracture surface, and (**b**) optical microscope image of the specimen edge at the fracture surface. Carbon fiber tows are present along 1-axis and load is applied along 2-axis.

All specimens exhibited a brittle failure mode with the fracture plane oriented perpendicular to the loading direction (i.e., along the direction of the carbon fiber tows), regardless of the strain rate (Figure 13). For the quasi-static specimens, the fracture surface was relatively smooth in appearance, and local observations revealed bare fibers and resin-rich regions (Figure 14a), indicating that the fracture plane comprised localized matrix cracks and fiber/matrix interfacial debonds. Observations through the entire specimen thickness revealed that the fracture surface propagated through the tows and not along the tow boundaries (Figure 14b). The fracture surfaces were similar for the specimens tested at higher strain rates; however, the appearance of the fracture surface was rough, and pullout of the supporting glass fiber yarns was observed (Figure 13b,c). The distinct local characteristics of the fracture surfaces for specimens tested under elevated strain rates may be due to a higher degree of local brittle crack propagation at the fracture plane caused by the increased stiffness of the matrix. Zeng [14] reported a 20% increase in the tensile modulus of the neat resin with an increase in strain rate over the range considered in this study.

### 3.2. Transverse Compression Tests

**Figure 15:**
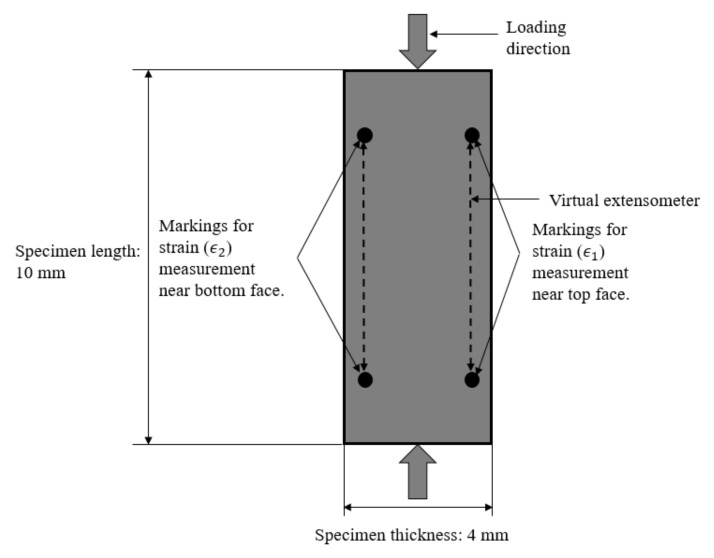
A schematic diagram showing the side view of a specimen used for the high rate (at 325 s^−1^) transverse compression testing. The dots were marked near the top and bottom faces of the specimen to obtain strains for computing the bending percentage.

**Figure 16:**
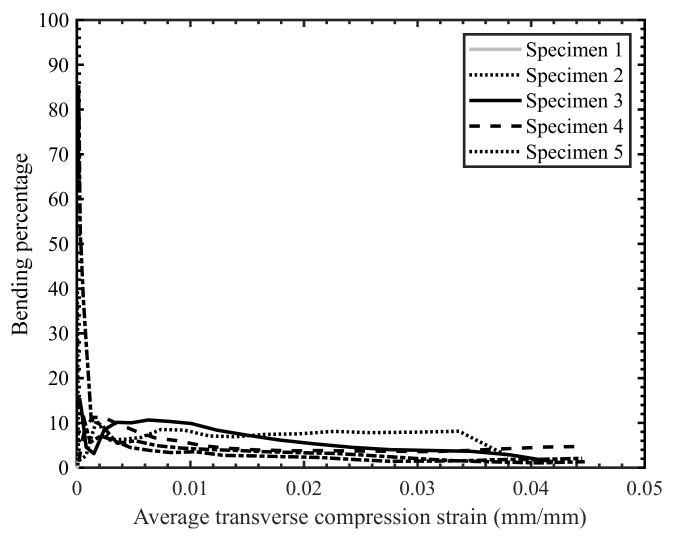
Bending percentage vs. average strain for the high strain rate transverse compression tests.

During uniaxial compression tests, flat specimens may excessively bend and fail prematurely due to the macro-buckling, rendering the data inaccurate, including the compressive strength. Therefore, for a compression test to be valid the percent bending should be within a limit of 10% as per ASTM D3410 [19], which can also be adopted for dynamic tests. The bending percentage is calculated as

(4) $$\mathrm{Bending}\;\mathrm{percentage}\;=\;\frac{\epsilon_{1}-\;\epsilon_{2}}{\epsilon_{1}+\epsilon_{2}}\cdot 100$$

in which $\epsilon_{1}$ and $\epsilon_{2}\;$ represent the strain values on the top and bottom faces of the specimen, respectively. In this study, the strain values on the specimen faces were determined by capturing the deformation of points located on the edge of the specimen near the specimen faces (Figure 15). As an example, for the high strain rate transverse compression tests, the resulting bending percentage was found to be 10% or lower (Figure 16). An additional benefit of capturing deformation of the specimen edge was that the fracture angle was also obtained.

**Figure 17:**
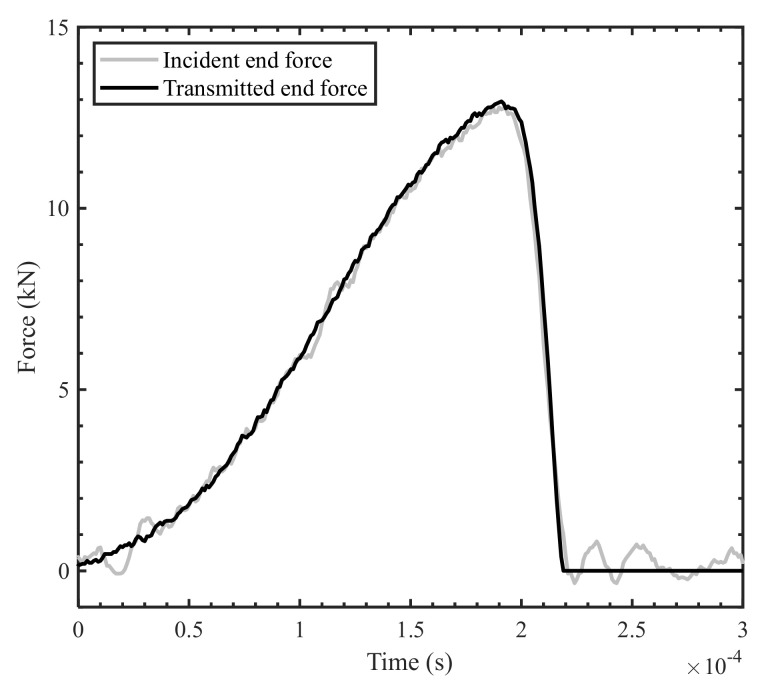
Force comparison at the incident and transmitted bar ends for a high strain rate transverse compression test.

**Figure 18:**
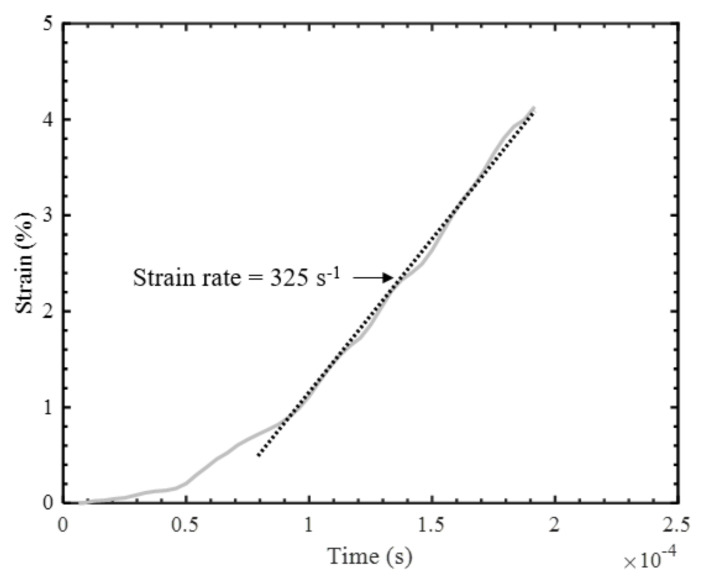
Strain variation during a high strain rate transverse compression test.

For the high strain rate tests conducted on the SHPB apparatus, it was also necessary to confirm that dynamic equilibrium was achieved and that the strain rate was nearly constant to ensure reliability of the data. After the initial loading period, the measured incident bar end and transmitted bar end forces were consistent throughout the duration of the tests within 10% (Figure 17), which indicates that dynamic equilibrium was established for all conducted tests. Furthermore, the strain was found to increase linearly with time after the initial loading period, allowing a nearly constant strain rate to be calculated on the basis of the slope of the linear region of the strain-time plot (Figure 18).

**Figure 19:**
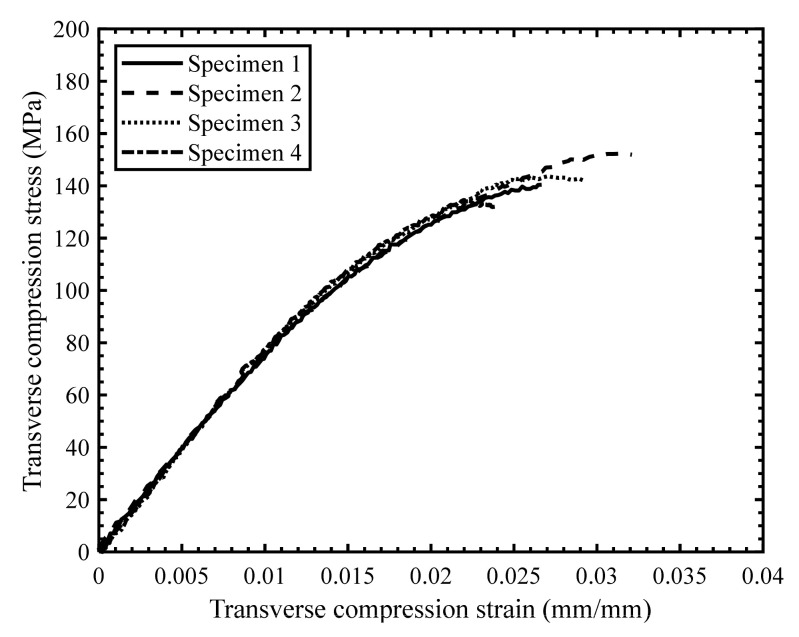
Stress–strain response for transverse compression tests with a strain rate of 0.003 s^−1^.

**Figure 20:**
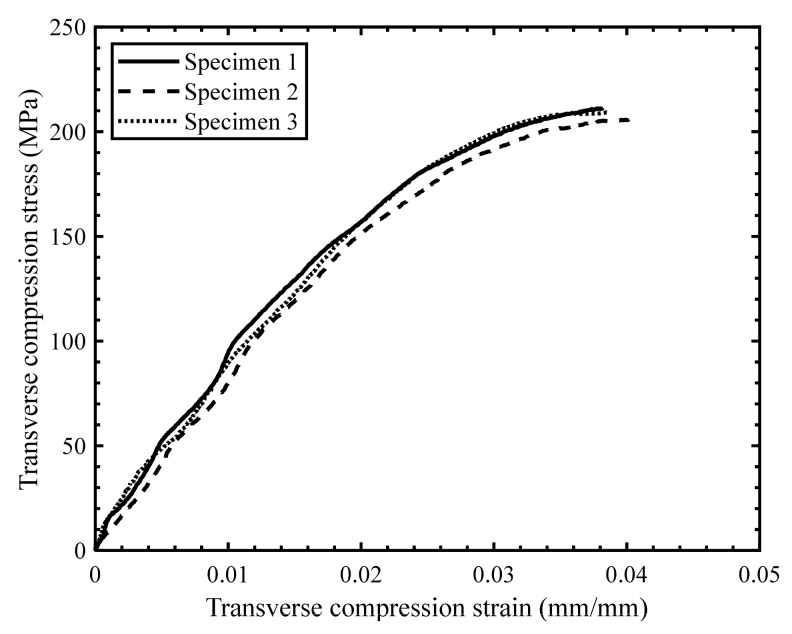
Stress–strain response for transverse compression tests with a strain rate of 0.2 s^−1^.

**Figure 21:**
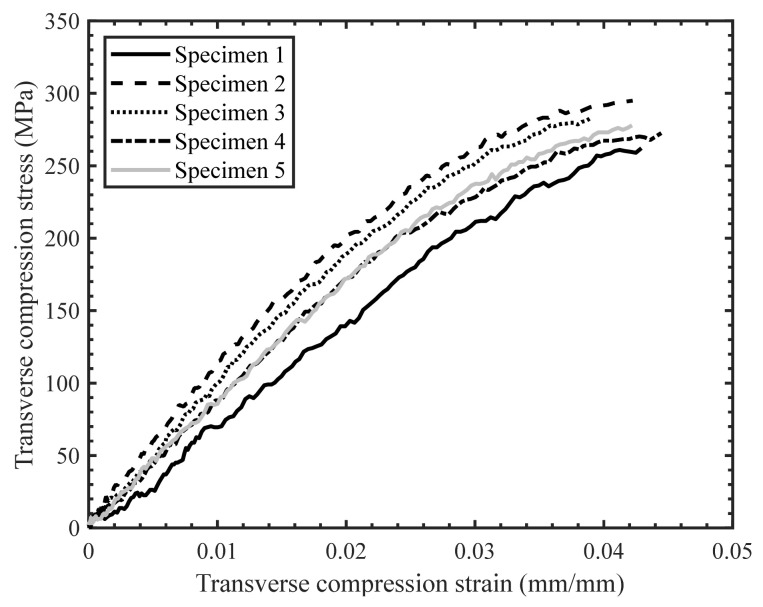
Stress–strain response for transverse compression tests with a strain rate of 325 s^−1^.

The scatter in the stress–strain response for the quasi-static (Figure 19) and intermediate strain rate tests (Figure 20) was found to be less than for the high strain rate tests (Figure 21), which was anticipated due to the characteristics of the SHPB test set-up in general and consistent with previous studies by Gilat and Seidt [20]. The specimens initially exhibited a linear stress–strain response at all strain rates; however, the stress–strain response became non-linear with increasing strain. The non-linearity in the transverse compressive stress–strain response may be due to the localized yielding in the matrix and/or the development of microcracks. Note that Zeng [14] reported significant non-linearity in the stress–strain response for the neat resin under compression and shear loading over a range of strain rates.

**Figure 22:**
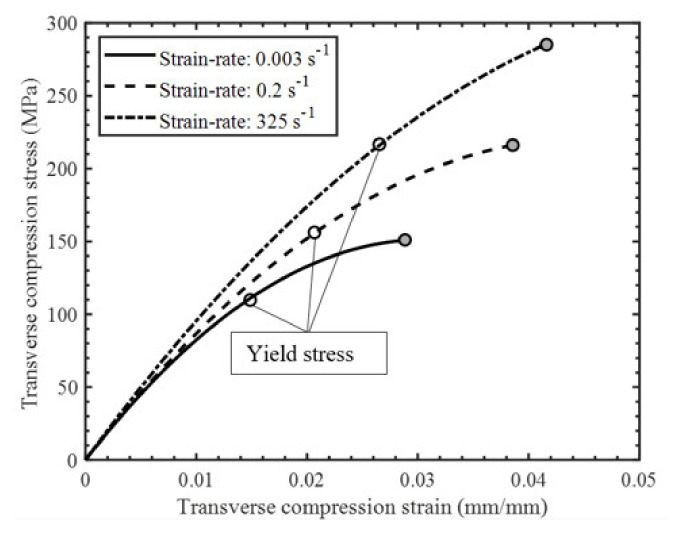
Representative transverse compression stress–strain response (second–order polynomial fit) for indicated strain rates.

**Figure 23:**
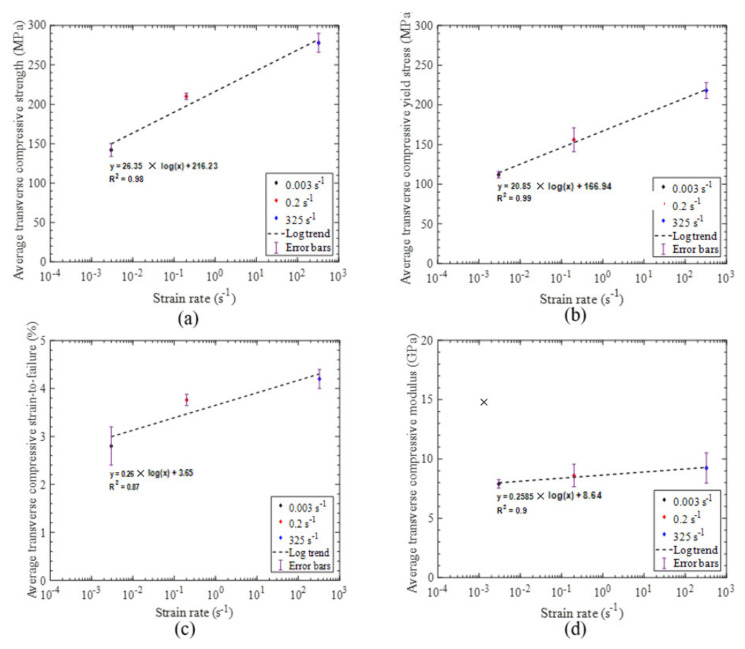
(**a**) Average transverse compressive strength versus strain rate. (**b**) Average transverse compressive yield stress versus strain rate. (**c**) Average transverse compressive strain–to–failure versus strain rate. (**d**) Average transverse compressive modulus versus strain rate with regression lines shown.

Comparing the stress–strain response for the quasi-static, intermediate, and high strain rate experiments (Figure 22) and the measured properties (Table 2), we found that strain rate appeared to have slight positive effect on the initial stiffness. However, the effects of strain rate were found to be significant on the yield stress (defined by the 0.2% offset method) and ultimate strength, with a 94% and 96% increase, respectively, over the range of strain rates considered. Similar trends for the effect of strain rate on the compressive elastic modulus, yield stress, and ultimate strength were also reported in previous studies on UD tape composites by Koerber et al. [6] and Schaefer et al. [4]. The strain-to-failure measured at intermediate and high strain rates was found to be higher than the strain-to-failure obtained at the quasi-static strain rate in the present study (Table 2), which is contrary to the trends in these values with strain rate reported in the other studies for UD tape composites [4,6]. The empirical relationships for the average transverse tensile strength, yield stress, strain-to-failure, and elastic modulus as a function of strain rate are established by fitting the data with a semi-logarithmic expression (Figure 23) that can be used to interpolate these properties at other strain rates.

**Figure 24:**
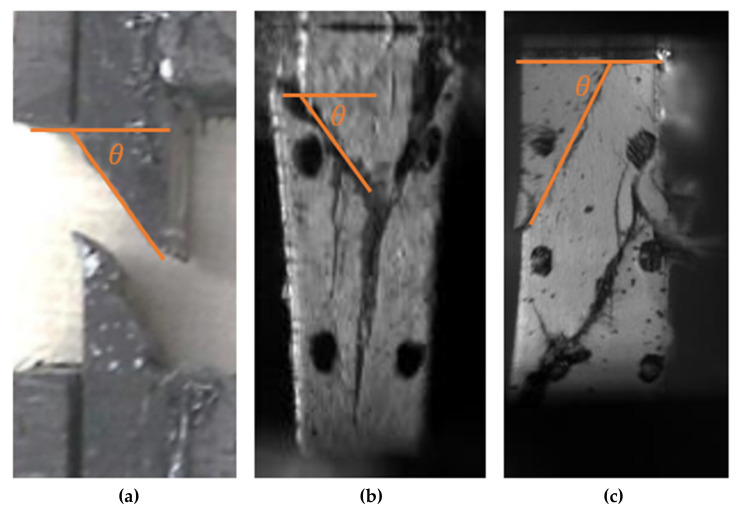
Images of failed transverse compression specimens at strain rates of (**a**) 0.003 s^−1^; (**b**) 0.2 s^−1^; (**c**) 325 s^−1^.

**Figure 25:**
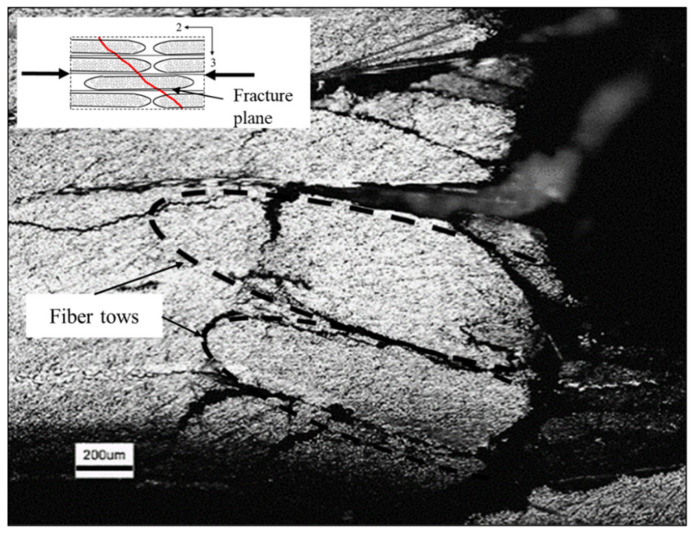
Quasi-static transverse compression test specimen optical microscope image of the edge at the fracture surface. The load is applied along the 2-axis.

Compressive loading along the transverse direction resulted in localized shear stress concentrations in the matrix and at the fiber/matrix interfaces, which subsequently resulted in the failure of the specimen along a plane biased to the loading direction (Figure 24). The average fracture angle (Table 2) for the quasi-static test specimens was found to be 53° (±1°), which is the value of 53° (±3°) reported by Koerber et al. [5] for UD carbon fiber/epoxy material [6]. The fracture angle for the high-rate tests was measured to be noticeably higher than that measured from the quasi-static tests. Most specimens tested at the high strain rate exhibited multiple biased cracks (Figure 24c). The presence of cracks towards both the incident and transmitted bar ends indicate that the deformation was homogenous, and specimens were under dynamic equilibrium conditions [21]. Cracks at multiple locations were also observed by Chen et al. [21] in the dynamic testing of neat polymers; thus, multiple biased cracks in the composite specimens are a consequence of high loading rates. Optical microscopy images of the edge of a quasi-static compression test specimen at the fracture surface revealed multiple secondary cracks in and around the carbon fiber tows (Figure 25). The reported angles are based on the first crack only since for subsequent loading the test specimens were no longer under pure transverse compression [10].

## 4. Conclusions

The effect of strain rate on the transverse tensile and compressive behaviour of a unidirectional non-crimp fabric (UD-NCF) carbon fiber/snap-cure epoxy composite was studied. Hydraulic testing frames were used for tests conducted under quasi-static and intermediate strains rates, while a split Hopkinson pressure bar (SHPB) apparatus was used for high strain rate tests. For the transverse tension deformation mode, the strength and strain-to-failure were found to increase by 16% and 20%, respectively, with increase in strain rate over the range of strain rates considered, while there was no statistically significant change in the elastic modulus. These collective results differ from those reported for carbon fiber/epoxy UD tape composites and are attributed to the distinct microstructure of the studied UD-NCF composite, which is an important finding of this study. The composite comprised tows with notably large resin-rich regions between the tows, which contributed to the observed strain rate dependency. Observations during the tests revealed that the fracture surface morphology was also influenced by the strain rate.

The effect of strain rate was more pronounced for the matrix-dominant transverse compression deformation mode, particularly the yield stress and strength, which is primarily attributed to the highly strain rate-dependent response of the resin. The neat resin was previously characterized and reported to have a significant strain rate dependency in compression and shear. The orientation of the through-thickness fracture plane was also found to increase with increasing strain rate.

The outcomes of this study have led to a deeper understanding of the strain rate-dependent deformation response and failure modes for a UD-NCF composite material under transverse tension and compression loading. Moreover, the material data set generated in this study has addressed a critical gap in the literature for UD-NCF composites and can be used to calibrate damage-based constitutive models and support the development of numerical models for crash simulations in future studies.

## Figures and Tables

**Table 1:** Mean transverse tensile properties of the UD-NCF composite [0]_7_ for indicated strain rates reported with standard deviation (STDV) and coefficient of variance (CV).

Strain Rate (s^−1^)	No. of Tests		Strength (MPa)	Strain-to-Failure (%)	Modulus (GPa) (Measured Range 0.15–0.35%)
2.2 ×10^−4^	5	Mean	62	0.81	8.4
		STDV	3.8	0.06	0.3
		CV (%)	6	7	4
0.1	4	Mean	66	0.88	7.8
		STDV	3	0.08	0.66
		CV (%)	4.6	9.1	8
16	4	Mean	72	0.98	8.8
		STDV	4	0.09	0.38
		CV (%)	5	9	4

**Table 2:** Mean transverse compressive properties of the UD-NCF composite [0]_11_ for indicated strain rates reported with standard deviation (STDV) and coefficient of variance (CV).

Strain Rate (s^−1^)	No. of Tests		Strength (MPa)	Yield Stress (MPa)	Strain-to-Failure (%)	Modulus (GPa) (Measured Range 0.15–0.35%)	Fracture Angle, *θ* (°)
0.003	4	Mean	142	112	2.8	7.9	53
		STDV	8	4	0.4	0.4	1
		CV (%)	6	4	13	4.6	2
0.2	3	Mean	210	156	3.8	8.6	55
		STDV	4	24	0.12	1.0	2
		CV (%)	2	15	3	11.0	3
325	5	Mean	278	218	4.2	9.2	60*
		STDV	12	21	0.2	1.3	2*
		CV (%)	5	10	5	15.9	1*

* Data from 4 specimens were used to calculate mean fracture angle.
